# Our Meanest Crime

**Published:** 1893-01

**Authors:** 


OUR MEANEST CRIME: A paper read at the Church Congress at Folkestone, England, 1892, by John H. Clarke, M. D. London: Victoria Street and International Society for the Protection of Animals from Vivisection, 1892.

This paper strongly opposes vivisection performed on animals and experiments on hospital patients. Dr. Clarke says, "Vivisection means tedious and difficult observations of animals after they have been dissected alive and whilst they are still living; and so complicated is the process that it is the rarest thing for two experimenters to be agreed about the results of the same experiment." And, again, "That the results of experiments on animals cannot be taken as any guide to what will happen if the same experiment be tried on man." The work of the physician is to cure diseases, and if not to cure them, to *mitigate the pains* they produce. No *scientific experiment* can be offered as an excuse for causing agony and unnecessary pain to a fellow-mortal or animal. The same scientists would denounce loudly the gamblers in a cock-pit for the *brutal* treatment of the fowls, who are frequently killed by *one blow* or thrust of the sharpened blade. But this cruelty cannot be compared with the learned man or men who dig with their sharpened blades deep into the vitality, causing unknown pain to our poor dumb and all suffering animals. The Church cannot but denounce this "Our Meanest Crime."

